# Foodborne Diseases Active Surveillance Network—2 Decades of Achievements, 1996–2015

**DOI:** 10.3201/eid2109.150581

**Published:** 2015-09

**Authors:** Olga L. Henao, Timothy F. Jones, Duc J. Vugia, Patricia M. Griffin, for the Foodborne Diseases Active Surveillance Network (FoodNet) Workgroup

**Affiliations:** Centers for Disease Control and Prevention, Atlanta, Georgia, USA (O.L. Henao, P.M. Griffin);; Tennessee Department of Health, Nashville, Tennessee, USA (T.F. Jones);; California Department of Public Health, Richmond, California, USA (D.J. Vugia)

**Keywords:** Foodborne Diseases Active Surveillance Network, FoodNet, foodborne infections, laboratory-based surveillance, bacteria, enteric infections, parasites, hemolytic uremic syndrome, Emerging Infections Program, EIP

## Abstract

The Foodborne Diseases Active Surveillance Network (FoodNet) provides a foundation for food safety policy and illness prevention in the United States. FoodNet conducts active, population-based surveillance at 10 US sites for laboratory-confirmed infections of 9 bacterial and parasitic pathogens transmitted commonly through food and for hemolytic uremic syndrome. Through FoodNet, state and federal scientists collaborate to monitor trends in enteric illnesses, identify their sources, and implement special studies. FoodNet’s major contributions include establishment of reliable, active population-based surveillance of enteric diseases; development and implementation of epidemiologic studies to determine risk and protective factors for sporadic enteric infections; population and laboratory surveys that describe the features of gastrointestinal illnesses, medical care–seeking behavior, frequency of eating various foods, and laboratory practices; and development of a surveillance and research platform that can be adapted to address emerging issues. The importance of FoodNet’s ongoing contributions probably will grow as clinical, laboratory, and informatics technologies continue changing rapidly.

## Abstract 2

FoodNet has provided a foundation for food safety policy and illness prevention since 1996.

During the late 1980s and early 1990s, recognizing inconsistencies inherent in passive national surveillance systems, epidemiologists at the Centers for Disease Control and Prevention (CDC) proposed creating a population-based active surveillance system to better measure the frequency of enteric infections and their effects on society. However, resources for these improvements were not available. Then, in late 1992 and early 1993, hamburger patties contaminated with *Escherichia coli* O157 caused 732 laboratory-confirmed infections and the deaths of 4 children. After this outbreak, the US Department of Agriculture (USDA) implemented a risk-based meat inspection system. Public health and regulatory officials needed a method to determine whether the changes made by regulatory agencies and the industry were followed by declines in infections. The outbreak had focused attention on the need for reliable data on the incidence of infections caused by enteric pathogens; changes in the incidence over time; and estimates of the actual numbers of illnesses, hospitalizations, and deaths they cause. Therefore, in 1995, with support from Food Safety and Inspection Service (FSIS) of the USDA, CDC established the Foodborne Diseases Active Surveillance Network (FoodNet), an active, population-based sentinel surveillance system. FoodNet monitors changes in the incidence of selected major bacterial and parasitic illnesses transmitted commonly by food, attributes illnesses to sources and settings, and estimates the total numbers of foodborne illnesses in the United States.

## Overview and Purpose

**Figure 1:**
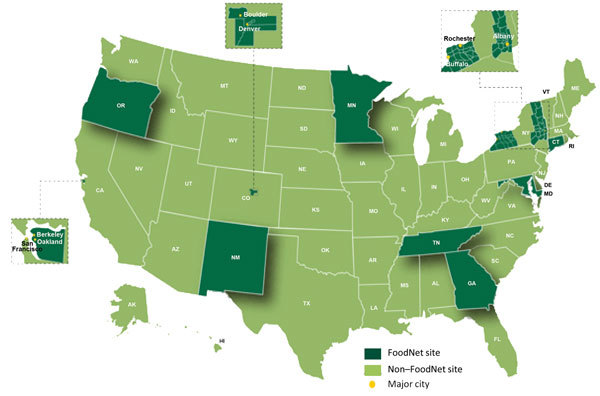
The Foodborne Diseases Active Surveillance Network Surveillance Area, United States, 2004–present.

FoodNet, a core part of CDC’s Emerging Infections Program, is a collaboration among CDC, 10 state health departments, USDA-FSIS, and the Food and Drug Administration (FDA). Over time, the surveillance area has grown to include ≈48 million persons (≈15% of the US population) in Connecticut, Georgia, Maryland, Minnesota, New Mexico, Oregon, and Tennessee and in selected counties in California, Colorado, and New York (*1*) (Figure 1). More information about the program and its activities is available at http://www.cdc.gov/FoodNet. The cost of funding for the surveillance sites and CDC, typically <$7 million per year, is dwarfed by the economic impact of the illnesses monitored. *Salmonella* infections alone cost ≈$3.6 billion each year in direct medical costs, productivity, and years of potential life lost (*2*).

The community of multidisciplinary FoodNet scientists collaborates to track infections transmitted commonly by food and to study the sources of infections. FoodNet’s annual report of confirmed infections caused by major pathogens, published within months of the end of each calendar year, is sometimes referred to as the foodborne illness “report card” for the nation. Public health officials, regulatory agencies, and industry use it to gauge progress in food safety and to determine when new policies and prevention efforts are needed (*3*).

FoodNet’s major contributions include the establishment of reliable, active population-based surveillance of enteric diseases; development and implementation of epidemiologic studies to determine risk and protective factors for sporadic enteric infections; population and laboratory surveys that describe the features of gastrointestinal illnesses, medical care–seeking behavior, food eating patterns, and laboratory practices; and development of a surveillance and research platform that can be adapted to address emerging issues (Table). It is the only US system focused on obtaining comprehensive information about sporadic infections caused by pathogens transmitted commonly through food.

**Table:** Major contributions of the Foodborne Diseases Active Surveillance Network (FoodNet), 1996–2015

Contribution	Specific contribution	Example of impact
Reliable active population-based surveillance of enteric diseases	FoodNet publishes incidence data for the previous year every spring. Rich database has comprehensive epidemiology and laboratory information about sporadic infections	Regulatory agencies evaluate their prevention efforts and change policies as a result of FoodNet data. Industry food safety executives use FoodNet data to inform policies. FoodNet data has been used to describe the epidemiology of infections caused by pathogens transmitted commonly through food in 162 publications. (More information is available at http://www.cdc.gov/foodnet/publications/index.html.)
Epidemiologic studies that determine risk and protective factors for sporadic enteric infections	A case–control study of *Listeria* infections showed that infection was associated with eating melons. Case–control studies of *Campylobacter* and *Salmonella* infections showed higher risk for infection among infants that had ridden in a shopping cart next to meat or poultry.	Because of study results, cantaloupe was added to *Listeria* initiative questionnaire, and this addition helped to more quickly identify cantaloupes as the source in the 2011 outbreak. As a result, some retail stores are now providing bags near the meat and poultry counters and are providing wipes for cleaning shopping carts.
Population and laboratory surveys that describe the features of gastrointestinal illnesses, medical care–seeking behavior, foods eaten, and laboratory practices	Estimates were made in 1999 and 2011 of the actual number of foodborne illnesses, including those not confirmed by a laboratory test.	The 2011 estimates were used to help determine the number of illnesses that could be attributed to each major food category. Regulatory agencies are using the latter estimates to guide prevention efforts.
Surveillance and research platform that can be adapted to address emerging issues	In 2008, as more clinical laboratories began adopting culture-independent diagnostic tests (CIDTs) for enteric pathogens, FoodNet responded by gathering data on enteric pathogens detected by these tests.	FoodNet worked with the Council of State and Territorial Epidemiologists to write a proposal to make *Campylobacter* infection diagnosed by either culture or CIDT a reportable condition nationwide. The proposal was approved in 2014, and reporting began in January 2015.

## Specific Activities and Selected Accomplishments

### Active Surveillance

FoodNet’s core activity is active surveillance for laboratory-confirmed bacterial infection caused by *Campylobacter*, *Listeria*, *Salmonella*, Shiga toxin–producing *Escherichia coli* (STEC) O157 and non-O157, *Shigella*, *Vibrio*, and *Yersinia* and parasitic infection caused by *Cryptosporidium* and *Cyclospora*. FoodNet does not track agents for which clinical laboratories do not routinely test (e.g., norovirus). As laboratory practices change, the surveillance system adapts. During the 2000s, culture-independent diagnostic tests (CIDTs) became commercially available to detect Shiga toxin. Recognizing that some laboratories might stop culturing for STEC O157, leading to a perceived decline in infections, FoodNet began surveillance for hemolytic uremic syndrome (HUS) as another way to track STEC O157 infections. Most cases of HUS are caused by STEC O157. As more clinical laboratories began using CIDTs to detect other pathogens, FoodNet responded by gathering data on laboratory practices and on pathogens detected by these tests and by encouraging reflex culturing of specimens that test positive (*4*).

FoodNet staff at each site receive reports of every identification of a pathogen under surveillance from clinical laboratories that conduct tests on patients’ specimens ordered by health care providers. They conduct periodic audits to ensure that all pathogens identified are reported (*3*). In 1999, to ensure the validity of data summarized across all sites, FoodNet developed and began tracking metrics related to reporting, a process unusual for CDC programs at that time. For each person with an infection, FoodNet staff collect demographic information and determine whether the person was hospitalized and whether he or she survived. In 2004, FoodNet began collecting data on whether the infection was part of an outbreak and whether the patient had traveled internationally.

FoodNet has the flexibility to conduct special surveillance projects in response to concerns about emerging infectious diseases, as when variant Creutzfeldt-Jakob disease emerged (*3*). In 2010, FoodNet conducted surveillance for *Cronobacter sakazakii* infections and found infection in all age groups, the highest rate of invasive infections in infants, and data suggesting that urine might be a more common site of infection than previously thought (*5*).

### Tracking Incidence and Changes Over Time

FoodNet tracks the incidence of infections and HUS to assess the effectiveness of measures aimed at preventing infections and to monitor progress toward national health goals. To measure changes over time and minimize the spurious effect of annual fluctuations, FoodNet has used 2 baseline periods of 3 consecutive years each. The first, 1996–1998, is the initial 3 years of surveillance; the second, 2006–2008, was used to develop the US Department of Health and Human Services Healthy People 2020 goals (*6*). In 2008, FoodNet began also reporting changes from the average annual incidence for the 3 years preceding the year of the report. In 2012, FoodNet began reporting a measure of overall change in the incidence of bacterial foodborne illness. This measure combines data for infections caused by the 6 bacterial pathogens monitored by the network for which >50% of illnesses are estimated to be transmitted by food (*7*). To account for variations in the surveillance area, FoodNet uses a main-effects log-linear Poisson (negative binomial) regression model to assess changes in incidence rates (*1*).

**Figure 2:**
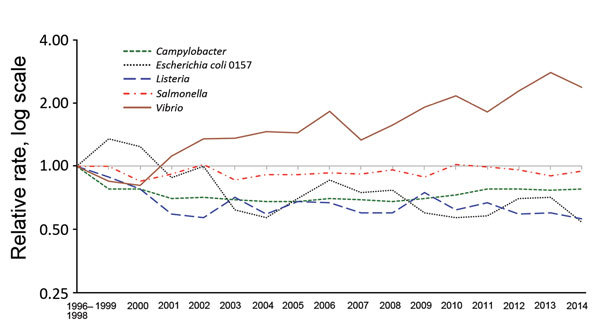
Relative rates of culture-confirmed infections with *Campylobacter*, *Escherichia coli* O157, *Listeria*, *Salmonella*, *Vibrio,* and *Yersinia* compared with 1996–1998 rates, Foodborne Diseases Active Surveillance Network, United States, 1996–2014. The position of each line indicates the relative change in the incidence of that pathogen compared with 1996–1998. The actual incidences of these infections cannot be determined from this graph. Data for 2014 are preliminary.

Each spring, FoodNet summarizes preliminary data and changes in incidence for the preceding year (Figure 2) in CDC’s Morbidity and Mortality Weekly Report. Public health officials, regulatory agencies, industry, and consumer groups use these data to assess the effect of food safety interventions (*3*). FoodNet has documented significant decreases in the incidence of *E. coli* O157 infections since 1996–1998 and in HUS since 2001, supporting other data indicating that regulatory and industry actions have made ground beef safer (*8*). FoodNet also has documented lack of significant change in the overall incidence of *Salmonella* infections and marked changes in some specific serotypes, indicating that efforts targeting specific serotypes are needed to decrease *Salmonella* infections. In response to these findings and to recent outbreaks, FSIS created performance standards mandating the upper limit of allowable *Salmonella* contamination of chicken parts (*8*). Poultry is also a major source of *Campylobacter* infections (*9*). In 2011, in response to FoodNet data showing little progress in reducing these infections, FSIS issued the first performance standards that limited the allowable contamination of chicken and turkey with *Campylobacter* (*10*).

FoodNet data are used to guide the development of and monitor progress toward national goals and health objectives, such as the US Department of Health and Human Services’ high priority goal to reduce *S. enterica* serotype Enteritidis infections from eggs after implementation of the Egg Safety Rule that was passed in 2009 (*6*). FoodNet data also are used to monitor progress on 7 illnesses included in the Healthy People National Health Objectives.

### Determining Sources and Outcomes of Infections

Understanding sources and settings of illnesses informs the development of recommendations, regulations, and interventions to reduce illnesses. FoodNet collects data to determine the relative importance of various routes of infection, including nonfood sources. Kendall et al. reported that, with wide variation by pathogen, 13% of persons infected with a pathogen monitored by FoodNet had recently traveled internationally; the authors identified regions to which travel carries the highest risk for illness (*11*). Hale et al. used data from FoodNet and other sources to estimate that 13% of illnesses caused by 7 enteric pathogens were attributable to contact with animals and their environments (*12*).

FoodNet has identified numerous risky food, environment, and animal exposures through case–control studies of sporadic *Campylobacter*, *Cryptosporidium*, *Listeria*, *Salmonella*, and STEC O157 infections, described in 19 journal articles (more information available at http://www.cdc.gov/FoodNet). It is currently conducting a case–control study of non-O157 STEC infections to assess risk factors and correlate virulence factors with symptoms. These studies have yielded rich data of long-term value. When the FDA needed data on sources of *S. enterica* serotype Enteritidis illnesses before the Egg Rule was implemented, Gu et al. reanalyzed data from an old FoodNet case–control study with a new method and determined that egg-related exposures had the highest attributable fraction (*13*). A case–control study of *Listeria* infections showed an unexpected association with eating melons (*14*). In response, CDC modified the *Listeria* Initiative questionnaire used to interview patients with *Listeria* infection. As a result, when Colorado detected a large *Listeria* outbreak, investigators already had information about cantaloupe consumption from many patients, and the melons were more quickly implicated and removed from the market (*15*). Case–control studies are resource-intensive and so are conducted infrequently. In 2014 FoodNet began routinely collecting exposure data from patients with some *Salmonella* infections and is exploring ways to use these data in models that attribute illnesses to sources.

### Population and Laboratory Surveys

FoodNet conducted 5 population surveys beginning in 1996, with only a 2-year gap before the last survey ended in 2007. In addition to obtaining data for estimating illnesses (described in the next section), the surveys asked participants how recently they ate selected foods. These data have been used for many analyses. Shiferaw et al. found a higher proportion of men reported eating pink hamburger and runny eggs, whereas a higher proportion of women ate fruits and vegetables (*16*). The population surveys have been used frequently in outbreak investigations. Epidemiologists compare frequencies of specific exposures reported by outbreak patients with those of a comparable population in the survey to quickly generate, confirm, or refute hypotheses about sources of illness. The ready availability of these data saves time over traditional methods of finding controls (e.g., by random-digit telephone calls, followed by interviews of persons reached who agree to participate) (*17*). During a 2012–2013 multistate outbreak of *S. enterica* serotype Heidelberg infections, 79% of patients reported eating chicken in the week before illness began, significantly higher than the 65% reported in the 2006–2007 FoodNet population survey (*18*). That finding, with other epidemiologic, laboratory, and traceback findings, helped link the outbreak to chicken from 1 producer. The population surveys also have provided a platform for obtaining information quickly in a crisis. When bovine spongiform encephalopathy emerged as a public health concern during the mid-2000s, questions about hunting practices, eating venison, and travel to countries in which bovine spongiform encephalopathy had been reported in animals were added to the 2006–2007 survey (*19*).

FoodNet conducts surveys of clinical laboratories to determine practices. By analyzing data from surveys conducted in 1995, 1997, and 2000, Voetsch et al. determined that variations in laboratory practice by site might explain some of the observed differences in the incidence of STEC O157 infection (*20*). A survey in 2005 found that adherence to recommendations for isolation and identification of *Campylobacter* varied substantially among laboratories (*21*). A survey in 2007 showed that most laboratories complied with recommendations for testing STEC O157 but not with recommendations for non-O157 STEC (*22*). The laboratory surveys provided essential information for estimating the true number of enteric infections (*23*). In 2012, because of rapidly changing clinical laboratory practices, FoodNet began conducting a survey annually. The 2014 survey showed that CIDT methods were used most often to detect *Campylobacter* and STEC (*4*).

### Estimating Actual Foodborne and Acute Gastrointestinal Illnesses, Hospitalizations, and Deaths

FoodNet data are central to estimating the numbers of US foodborne illnesses, hospitalizations, and deaths (*23*,*24*). Regulatory agencies and lawmakers use these estimates to help decide how to allocate resources for prevention. Mead et al. published the first estimates in 1999 in Emerging Infectious Diseases (*24*) using early active surveillance data from FoodNet and data from other sources. By 2010, this article was the most frequently cited article published in this journal. After these estimates were published, FoodNet began addressing data gaps and developing improved methods, resulting in revised comprehensive estimates published by Scallan et al. in 2011 (*23*). Major improvements resulted from the availability of data from >5 times more respondents to population surveys and more detailed information about illnesses reported in those surveys. For both the 1999 and the 2011 estimates, these surveys provided key data on the severity of illnesses, medical care–seeking behavior, and specimen submission. These data and data from FoodNet surveys of laboratories were used to estimate the total number of illnesses for every reported laboratory-confirmed illness of each pathogen. The surveys also provided essential information about the rate of acute gastroenteritis illnesses, which was used to estimate illnesses caused by viral pathogens and by unknown agents (*25*). An important advancement in the 2011 article was separate estimation of the numbers of illnesses acquired domestically and during international travel, enabled by FoodNet’s collection of information about recent international travel. These new foodborne illness estimates formed the basis for a ground-breaking analysis estimating the number of illnesses attributed to specific food categories (*26*).

### Other Contributions

Linking FoodNet to the National Antimicrobial Resistance Monitoring System (NARMS) has expanded the impact of both surveillance systems. A FoodNet case–control study linked fluoroquinolone-resistant *Campylobacter* infections with eating poultry at a commercial establishment and with international travel (*27*). Data from this study contributed to the body of evidence that led FDA to withdraw approval for the use of fluoroquinolones in poultry (*28*). Krueger et al. conducted a joint FoodNet–NARMS study that showed bloodstream infection was more common among patients infected with resistant than susceptible *Salmonella* strains (*29*). By linking FoodNet and NARMS data, Shiferaw et al. found that *Shigella* isolates from Hispanics and recent international travelers were more likely than other isolates to be resistant to trimethoprim/sulfamethoxazole (*30*). In another study linking these 2 databases, O’Donnell et al. found that two thirds of persons with *S. enterica* serotype Enteritidis infections resistant to nalidixic acid had recently traveled internationally (*31*).

The ability to geocode FoodNet surveillance data and link it to census data has increased FoodNet’s ability to examine health disparities. By geocoding *Campylobacter* cases and linking to census tract socioeconomic status (SES) measures, Bemis et al. found the incidence of campylobacteriosis in Connecticut increased as neighborhood SES increased except among children <10 years old, for whom incidence increased as SES decreased (*32*).

The benefits of FoodNet for public health are far-reaching. As experts in surveillance, FoodNet site epidemiologists are often leaders in conducting multistate outbreak investigations, many of which result in industry or regulatory changes that make food safer. Examples include an outbreak of *Salmonella* infections linked to pot pies (*33*), an outbreak of *E. coli* O157 infections linked to spinach (*34*), and the outbreak of *Listeria* infections linked to cantaloupe (*15*). FoodNet site personnel also train local public health nurses, epidemiologists, sanitarians, and laboratorians about foodborne disease surveillance, outbreak detection, investigation, and response. Training helps local public health professionals recognize outbreaks and maintain skills and knowledge needed to respond appropriately.

FoodNet’s influence reaches beyond the United States. Australia’s OZFoodNet and FoodNet-Canada have modeled some of their activities on FoodNet surveillance, and FoodNet staff have collaborated with scientists from other countries to compare the prevalence of diarrheal illness (*35*). FoodNet scientists have been active in the World Health Organization Global Foodborne Infections Network, which works to enhance the capacity of countries to detect, respond to, and prevent foodborne and other enteric infections.

## Challenges and the Future

The importance of FoodNet’s ongoing contributions toward developing epidemiologic methods for assessing diseases transmitted commonly by food likely will grow as clinical, laboratory, and informatics technologies continue changing at a rapid pace. Recent and ongoing advances in CIDTs and molecular diagnostics affect FoodNet surveillance. FoodNet’s responsiveness to this changing landscape is informing ongoing modifications of national surveillance definitions that CDC and all US states use.

For the near future, although CIDTs serve clinical needs, bacterial isolates remain essential for the molecular subtyping and antimicrobial drug susceptibility testing needed for epidemiologic monitoring, outbreak detection, and public health investigations. Public health laboratories in FoodNet states could become key sites for maintaining public health access to isolates of enteric pathogens obtained by reflex culturing after a positive CIDT. Maintaining access to traditional laboratory methods also is necessary to validate and interpret new technologies. Traditional laboratory methods might be needed to help evaluate the significance of detection using highly sensitive genetic techniques of multiple pathogens in a single specimen. Because FoodNet surveillance is built on clinical and public health laboratory diagnosis, laboratories must have the resources required to meet surveillance needs.

As laboratories adopt whole-genome sequencing to identify and characterize enteric pathogens, the ability to identify subtypes associated with particular reservoirs and particular food sources will increase. Detailed epidemiologic data on exposures of ill persons will be needed to make these associations. At this time, although many state and local health departments obtain exposure information, FoodNet surveillance captures only a limited amount. Obtaining more will involve duplicate data entry or designing information technology systems that can interface with a variety of databases housed at local and state health departments and at CDC.

The advent of CIDTs offers opportunities to conduct surveillance for enteric pathogens not monitored now. Some CIDTs can detect enterotoxigenic *E. coli* infection, which is an important cause of diarrhea in returning travelers and has caused domestic outbreaks (*36*). The large proportion of the US food supply that is imported, including many fruits and vegetables that are eaten raw, provides opportunities for exposure to pathogens from all over the world. Expanding surveillance to enterotoxigenic *E. coli* and other pathogens, after clinical laboratories begin detecting them, could lead to greater insight into the causes and sources of enteric infections in the United States and abroad.

FoodNet population surveys have proven valuable as sources of data about rates and severity of acute gastrointestinal illnesses and medical care–seeking and about food eaten and other exposures among well persons in the community (*16*,*37*,*38*). The lack of a population survey after 2007 means that data needed to update estimates of the impact of illness are not available. Although the frequency that various foods are eaten may have changed, these data are still used because up-to-date data are not readily available from other sources. FoodNet is working with its partners to find ways to fund and conduct more frequent surveys.

Awareness is increasing of the need to combat antimicrobial drug resistance. Four of the 18 threats that CDC reported in Antibiotic Resistance Threats in the United States, 2013, are tracked in FoodNet (*39*).The National Strategy for Combating Antibiotic Resistant Bacteria, announced in 2014 (*40*), aims to slow the emergence of resistant bacteria and prevent the spread of resistant infections. Surveillance data are needed to determine whether strategies to preserve the effectiveness of antimicrobial drugs are working and whether new threats are emerging. FoodNet’s longstanding collaboration with NARMS likely will increase further to meet this need.

CDC’s information technology method for obtaining surveillance data from FoodNet sites needs updating, including developing the ability to obtain and analyze data by person and to interface with national surveillance systems. FoodNet creates a separate record for each illness diagnosed by the detection of a pathogen; ways to link the record to the ill person are needed to determine whether a person has a co-infection or has sequential illnesses with several pathogens. The existence of a variety of methods for reporting infections to CDC is an ongoing challenge for state health officials. The use of different identifiers for information about the same isolate or illness reported to various CDC surveillance systems (e.g., FoodNet, PulseNet, NARMS) is an obstacle to fully understanding the features of reported infections. FoodNet staff will be engaged in efforts to ensure that national surveillance systems are designed to meet the needs of both states and CDC and that they enable accurate and timely analysis and release of FoodNet data.

The widespread growth of electronic health records in the clinical community presents challenges and opportunities for public health. CDC’s Emerging Infections Program is developing informatics capacity to incorporate data streams from electronic health records, electronic laboratory reporting, and other sources of “big data” (e.g., administrative claims data, social media). FoodNet databases at the sites and CDC are increasingly conforming to national data standards, which will facilitate linking to meaningful use of certified electronic health records technology. Rapid access to clinical data will improve surveillance and epidemiologic studies. Informatics capacity is essential for linking FoodNet surveillance data with geographic information systems and other public health databases (e.g., hospital discharge data, vital statistics, NARMS surveillance data). Lessons learned from pilot projects conducted at FoodNet sites could provide an important foundation for developing public health informatics infrastructure nationally. Through multiagency partnership and collaboration, FoodNet has helped improve food safety in the United States in multiple ways. The surveillance network, which began as a project, provides key data for public health analyses and decision-making, and has become an integral part of CDC’s work. FoodNet has matured and transformed over the last 20 years and continues to evolve. Changes in diagnostic practices that affect surveillance and the need for more detailed and precise information about the major sources of infections and how they change over time are just a few of the issues FoodNet will address during the next decade.

## References

[1] Henao OL, Scallan E, Mahon B, Hoekstra RM. Methods for monitoring trends in the incidence of foodborne diseases: Foodborne Diseases Active Surveillance Network 1996–2008. Foodborne Pathog Dis. 2010;7:1421–6. 10.1089/fpd.2010.0629 20617933

[2] Hoffman S, Maculloch B, Batz M. Economic burden of major foodborne illnesses acquired in the United States. Economic Information Bulletin no. 140. May 2015 [cited 2015 Jun 9]. http://www.ers.usda.gov/media/1837791/eib140.pdf

[3] Scallan E. Activities, achievements, and lessons learned during the first 10 years of the Foodborne Diseases Active Surveillance Network: 1996–2005. Clin Infect Dis. 2007;44:718–25. 10.1086/511648 17278067

[4] Iwamoto M, Huang JY, Cronquist AB, Medus C, Hurd S, Zansky S, Bacterial enteric infections detected by culture-independent diagnostic tests—FoodNet, United States, 2012–2014. MMWR Morb Mortal Wkly Rep. 2015;64:252–7 . 25763878 PMC5779603

[5] Patrick ME, Mahon BE, Greene SA, Rounds J, Cronquist A, Wymore K, Incidence of *Cronobacter* spp. infections, United States, 2003–2009. Emerg Infect Dis. 2014;20:1520–3 . 10.3201/eid2009.140545 25148394 PMC4178417

[6] Centers for Disease Control and Prevention. Incidence and trends of infection with pathogens transmitted commonly through food—Foodborne Diseases Active Surveillance Network, 10 U.S. sites, 1996–2012. MMWR Morb Mortal Wkly Rep. 2013;62:283–7 . 23594684 PMC4604974

[7] Henao OL, Crim SM, Hoekstra RM. Calculating a measure of overall change in the incidence of selected laboratory-confirmed infections with pathogens transmitted commonly through food in the Foodborne Diseases Active Surveillance Network (FoodNet), 1996–2010. Clin Infect Dis. 2012;54(Suppl 5):S418–20. 10.1093/cid/cis244 22572663

[8] Crim SM, Griffin PM, Tauxe R, Marder EP, Gilliss D, Cronquist AB, Preliminary incidence and trends of infection with pathogens transmitted commonly through food—Foodborne Diseases Active Surveillance Network, 10 US sites, 2006–2014. MMWR Morb Mortal Wkly Rep. 2015;64:495–9 . 25974634 PMC4584825

[9] Friedman CR, Hoekstra RM, Samuel M, Marcus R, Bender J, Shiferaw B, Risk factors for sporadic *Campylobacter* infection in the United States: a case–control study in FoodNet sites. Clin Infect Dis. 2004;38(Suppl 3):S285–96. 10.1086/381598 15095201

[10] US Department of Agriculture-Food Safety Inspection Service. New performance standards for *Salmonella* and *Campylobacter* in young chicken and turkey slaughter establishments: response to comments and announcement of implementation schedule. Fed Regist. 2011;76:15282–90 [cited 2015 Apr 2]. https://www.federalregister.gov/articles/2011/03/21/2011-6585/new-performance-standards-for-salmonella-and-campylobacter-in-young-chicken-and-turkey-slaughter

[11] Kendall ME, Crim S, Fullerton K, Han PV, Cronquist AB, Shiferaw B, Travel-associated enteric infections diagnosed after return to the United States, Foodborne Diseases Active Surveillance Network (FoodNet), 2004–2009. Clin Infect Dis. 2012;54(Suppl 5):S480–7. 10.1093/cid/cis052 22572673

[12] Hale CR, Scallan E, Cronquist AB, Dunn J, Smith K, Robinson T, Estimates of enteric illness attributable to contact with animals and their environments in the United States. Clin Infect Dis. 2012;54(Suppl 5):S472–9. 10.1093/cid/cis051 22572672

[13] Gu W, Vieira AR, Hoekstra RM, Griffin PM, Cole D. Use of random forest to estimate population attributable fractions from a case–control study of *Salmonella enterica* serotype Enteritidis infections. Epidemiol Infect. 2015;Feb:1–9; Epub ahead of print . 25672399 10.1017/S095026881500014X PMC9151037

[14] Varma JK, Samuel MC, Marcus R, Hoekstra RM, Medus C, Segler S, *Listeria monocytogenes* infection from foods prepared in a commercial establishment: a case–control study of potential sources of sporadic illness in the United States. Clin Infect Dis. 2007;44:521–8. 10.1086/509920 17243054

[15] McCollum JT, Cronquist AB, Silk BJ, Jackson KA, O’Connor KA, Cosgrove S, Multistate outbreak of listeriosis associated with cantaloupe. N Engl J Med. 2013;369:944–53. 10.1056/NEJMoa1215837 24004121

[16] Shiferaw B, Verrill L, Booth H, Zansky SM, Norton DM, Crim S, Sex-based differences in food consumption: Foodborne Diseases Active Surveillance Network (FoodNet) population survey, 2006–2007. Clin Infect Dis. 2012;54(Suppl 5):S453–7. 10.1093/cid/cis247 22572669

[17] Sheth AN, Hoekstra M, Patel N, Ewald G, Lord C, Clarke C, A national outbreak of *Salmonella* serotype Tennessee infections from contaminated peanut butter: a new food vehicle for salmonellosis in the United States. Clin Infect Dis. 2011;53:356–62. 10.1093/cid/cir407 21810748

[18] Centers for Disease Control and Prevention. Outbreak of *Salmonella* Heidelberg infections linked to a single poultry producer—13 states, 2012–2013. MMWR Morb Mortal Wkly Rep. 2013;62:553–6 . 23842445 PMC4604944

[19] Abrams JY, Maddox RA, Harvey AR, Schonberger LB, Belay ED. Travel history, hunting, and venison consumption related to prion disease exposure, 2006–2007 FoodNet Population Survey. J Am Diet Assoc. 2011;111:858–63. 10.1016/j.jada.2011.03.015 21616198

[20] Voetsch AC, Kennedy MH, Keene WE, Smith KE, Rabatsky-Ehr T, Zansky S, Risk factors for sporadic Shiga toxin–producing *Escherichia coli* O157 infections in FoodNet sites, 1999–2000. Epidemiol Infect. 2007;135:993–1000. 10.1017/S0950268806007564 17147834 PMC2870643

[21] Hurd S, Patrick M, Hatch J, Clogher P, Wymore K, Cronquist AB, Clinical laboratory practices for the isolation and identification of *Campylobacter* in Foodborne Diseases Active Surveillance Network (FoodNet) sites: baseline information for understanding changes in surveillance data. Clin Infect Dis. 2012;54(Suppl 5):S440–5. 10.1093/cid/cis245 22572667

[22] Hoefer D, Hurd S, Medus C, Cronquist A, Hanna S, Hatch J, Laboratory practices for the identification of Shiga toxin-–roducing *Escherichia coli* in the United States, FoodNet sites, 2007. Foodborne Pathog Dis. 2011;8:555–60. 10.1089/fpd.2010.0764 21186994

[23] Scallan E, Hoekstra RM, Angulo FJ, Tauxe RV, Widdowson MA, Roy SL, Foodborne illness acquired in the United States—major pathogens. Emerg Infect Dis. 2011;17:7–15. 10.3201/eid1701.P11101 21192848 PMC3375761

[24] Mead PS, Slutsker L, Dietz V, McCaig LF, Bresee JS, Shapiro C, Food-related illness and death in the United States. Emerg Infect Dis. 1999;5:607–25. 10.3201/eid0505.990502 10511517 PMC2627714

[25] Scallan E, Griffin PM, Angulo FJ, Tauxe RV, Hoekstra RM. Foodborne illness acquired in the United States—unspecified agents. Emerg Infect Dis. 2011;17:16–22. 10.3201/eid1701.P21101 21192849 PMC3204615

[26] Painter JA, Hoekstra RM, Ayers T, Tauxe RV, Braden CR, Angulo FJ, Attribution of foodborne illnesses, hospitalizations, and deaths to food commodities by using outbreak data, United States, 1998–2008. Emerg Infect Dis. 2013;19:407–15. 10.3201/eid1903.111866 23622497 PMC3647642

[27] Kassenborg HD, Smith KE, Vugia DJ, Rabatsky-Ehr T, Bates MR, Carter MA, Fluoroquinolone-resistant *Campylobacter* infections: eating poultry outside of the home and foreign travel are risk factors. Clin Infect Dis. 2004;38(Suppl 3):S279–84. 10.1086/381597 15095200

[28] Nelson JM, Chiller TM, Powers JH, Angulo FJ. Fluoroquinolone-resistant *Campylobacter* species and the withdrawal of fluoroquinolones from use in poultry: a public health success story. Clin Infect Dis. 2007;44:977–80. 10.1086/512369 17342653

[29] Krueger AL, Greene SA, Barzilay EJ, Henao O, Vugia D, Hanna S, Clinical outcomes of nalidixic acid, ceftriaxone, and multidrug-resistant nontyphoidal salmonella infections compared with pansusceptible infections in FoodNet sites, 2006–2008. Foodborne Pathog Dis. 2014;11:335–41. 10.1089/fpd.2013.1642 24617446

[30] Shiferaw B, Solghan S, Palmer A, Joyce K, Barzilay EJ, Krueger A, Antimicrobial susceptibility patterns of *Shigella* isolates in Foodborne Diseases Active Surveillance Network (FoodNet) sites, 2000–2010. Clin Infect Dis. 2012;54(Suppl 5):S458–63. 10.1093/cid/cis230 22572670

[31] O'Donnell AT, Vieira AR, Huang JY, Whichard J, Cole D, Karp BE. Quinolone-resistant *Salmonella enterica* serotype Enteritidis infections associated with international travel. Clin Infect Dis. 2014;59:e139–41. 10.1093/cid/ciu505 24973311

[32] Bemis K, Marcus R, Hadler JL. Socioeconomic status and campylobacteriosis, Connecticut, USA, 1999–2009. Emerg Infect Dis. 2014;20:1240–2 . 24964129 10.3201/eid2007.131333 PMC4073850

[33] Mody RK, Meyer S, Trees E, White PL, Nguyen T, Sowadsky R, Outbreak of *Salmonella enterica* serotype I 4,5,12:i:- infections: the challenges of hypothesis generation and microwave cooking. Epidemiol Infect. 2014;142:1050–60 . 10.1017/S0950268813001787 23916064 PMC9151130

[34] Centers for Disease Control and Prevention. Ongoing multistate outbreak of *Escherichia coli* serotype O157:H7 infections associated with consumption of fresh spinach—United States, September 2006. MMWR Morb Mortal Wkly Rep. 2006;55:1045–6 . 17008868

[35] Scallan E, Majowicz S, Hall G, Banerjee A, Bowman C, Daly L, Prevalence of diarrhoea in the community in Australia, Canada, Ireland, and the United States. Int J Epidemiol. 2005;34:454–60. 10.1093/ije/dyh413 15659464

[36] Dalton CB, Mintz ED, Wells JG, Bopp CA, Tauxe RV. Outbreaks of enterotoxigenic *Escherichia coli* infection in American adults: a clinical and epidemiologic profile. Epidemiol Infect. 1999;123:9–16. 10.1017/S0950268899002526 10487636 PMC2810723

[37] Patrick ME, Mahon BE, Zansky SM, Hurd S, Scallan E. Riding in shopping carts and exposure to raw meat and poultry products: prevalence of, and factors associated with, this risk factor for *Salmonella* and *Campylobacter* infection in children younger than 3 years. J Food Prot. 2010;73:1097–100 . 20537266 10.4315/0362-028x-73.6.1097

[38] Scallan E, Jones T, Cronquist A, Thomas S, Frenzen P, Hoefer D, Factors associated with seeking medical care and submitting a stool sample in estimating the burden of foodborne illness. Foodborne Pathog Dis. 2006;3:432–8. 10.1089/fpd.2006.3.432 17199525

[39] Centers for Disease Control and Prevention. Antibiotic resistance threats in the United States, 2013 [cited 2015 Apr 2]. http://www.cdc.gov/drugresistance/threat-report-2013/index.html

[40] President’s Council of Advisors on Science and Technology. National strategy for combating antibiotic-resistant bacteria. Sep 2014 [cited 2015 Apr 2]. https://www.whitehouse.gov/sites/default/files/docs/carb_national_strategy.pdf

